# Mining for Posttraumatic Growth (PTG) in Sexual Minority Women Who Survive Intimate Partner Violence: A Conceptual Perspective

**DOI:** 10.3390/bs8090077

**Published:** 2018-08-28

**Authors:** Elisabeth Counselman-Carpenter, Alex Redcay

**Affiliations:** 1 Department of Social Work, Southern Connecticut State University, New Haven, CT 06515, USA; 2 School of Social Work, Millersville University, Millersville, PA 17551, USA; alexredcay@gmail.com

**Keywords:** Post-traumatic growth, intimate partner violence, sexual minority women

## Abstract

This theoretical paper explores the need to use posttraumatic growth (PTG) as a framework when studying sexual minority women (SMW) who are survivors of intimate partner violence (IPV) to examine the relationship between risk factors such as stress, anxiety and alcohol use and to understand the role of protective factors through mining for the presence of posttraumatic growth (PTG). Despite a call for continued research in this highly vulnerable population, representative studies of SMW and PTG remain extremely limited. Research that examines the relationship between IPV, behavioral health issues, and posttraumatic growth would provide the opportunity to develop tailored intervention models and opportunities for program development to decrease isolation and increase factors of posttraumatic growth. In particular, the impact of how interpersonal relationships as potential mediators and/or outcomes of posttraumatic growth (PTG) needs to be explored more thoroughly. PTG is a valuable framework for vulnerable populations such as sexual minority women because it focuses on how transformative change may result from traumatic experiences such as surviving IPV.

## 1. Literature Review

### 1.1. Understanding Intimate Partner Violence and Sexual Minority Women

Intimate partner violence (IPV) is a significant public health issue and a prevalent social problem with up to 1.5 million women experiencing IPV in the United States in their lifetime, and upwards of 37% of women survive IPV in Southeast Asian, Eastern Mediterranean, and African countries [1,2,3]. In the United States, one in four women will experience severe physical violence from an intimate partner, one in ten women will be raped by an intimate partner, and nearly 50 percent will experience psychological aggression from an intimate partner in their lifetime [4]. 

IPV has been shown to cause increased mental health struggles including depression and anxiety, substance use and dependence disorders and Post Traumatic Stress Disorder (PTSD) [5,6]. However, most of the research conducted on IPV uses a heterocentric lens and focuses on heterosexual relationships. Risk factors increase exponentially when considering multiple systemic stressors and a history of historical trauma towards sexual minority women (SMW). Researchers who explore IPV in same-sex relationships have shared diverse findings. Frankland and Brown [7] found that levels of coercive control and patterns of violence in same-sex relationships are similar to categories identified in research with heterosexual couples, but did not look exclusively at women, while Gehring and Vaske [8] found that IPV in same-sex relationships led to increased levels of depressive symptoms and higher levels of involvement in violently delinquent behavior. Other theorists have explored more mezzo factors such as Sorensen and Thomas [9] who investigated societal views towards IPV in same-sex relationships and Baker et al. [10] who examined how cultural and systemic factors can be explored when gender biases are removed by looking at same-sex IPV.

### 1.2. Minority Stress Theory as a Theoretical Lens

One of the most frequently cited theories when researching or developing programs for sexual minorities is Meyer’s [11] Minority Stress Theory model. Minority stress theory states that those with marginalized sexual identities can have increased physical, mental and behavioral health outcomes [12]. Early research typically focused on both males and females in the LGB community, with only a recent focus of understanding gender-specific differences of minority stress [13]. The phrase ‘sexual minority stress’ currently refers to the experience of anyone who identifies as a sexual minority including men, women, and non-binary persons. However, there are unique features to the experience of sexual minority women including both internal stressors such as internalized homophobia (also known as internalized heterosexism), external life-events, perceived external stress and dual (multiple) stigmatization as women, as sexual minorities and the particular vulnerability to the intersection of other marginalized identities such as race that remain unexplored in the current research [12,14,15].

Sexual minority women (SMW) experience increased discrimination, stress and victimization more than non-minority women, which then is typically associated with even higher rates of mental health issues, particularly depression and anxiety and subsequent risk for substance use as a coping mechanism [12,16,17,18,19,20]. Risk factors continue to increase when taking into consideration geographic locale as research demonstrates that the more rural geographic area in which a sexual minority person resides, the greater the risk for health disparities [21] and lower use of health care and preventative services, including behavioral health supports [22].

Findings for SMW when considering IPV exclusively remains mixed in terms of prevalence as compared to heterosexual women. While we understand that general risk factors are higher for SMW, it is unclear as to whether or not the phenomenon of intimate partner violence actually occurs more in same-sex relationships. Some studies demonstrate rates of 25–50 percent in partnered lesbian relationships [23,24], some demonstrate lower rates of IPV in partnered lesbian relationships than in heterosexual relationships [25] and others showing rates upwards of 75 percent (15 percent) higher than heterosexual women [3,26]. In general, a thorough understanding of the prevalence of rates of IPV in same-sex relationships is challenged by a number of factors that include difficulty securing representative samples of queer women, difficulty or assumptions made between the perpetrator and the abuser, a general underreporting of the abuse, and a lack of a commonly agreed-upon definition of partner abuse [25,27]. The greatest levels of risk within the sexual minority community appear to be for bisexual women, with transwomen remaining significantly underrepresented in the literature [26]. This confusion regarding the occurrence of IPV in relationships of SMW is often due to study design as well as the heterocentric design of instruments [28,29].

## 2. Measurements

There are many assessment tools that measure some form of violence and abuse but few that do so specifically for SMW. General research challenges with IPV include multiple sampling issues and the timing of data collection when exploring this population due to where the data is gathered, such as short-term emergency housing or while the respondent is completing domestic violence programming, and there are significant challenges of follow-up due to frequent moves and loss of contact with various networks often due to ongoing safety issues [30,31,32]. 

However, specific to SMW, in addition to timing and sampling challenges, there are multiple issues with the measurement tools used to gather the data. Most assessments are lacking because of the heterocentric design of the instruments, which generally assume a heterosexual relationship and that the female is the victim. Typically, instruments that gather information about the experience of intimate partner violence use gendered language such as asking about one’s boyfriend or husband. In addition, the nature of the power and control within relationships is different among SMW. For example, one lesbian partner may threaten to “out,” or disclose the sexual orientation of the other lesbian partner at work. Finally, instruments do not typically gather data on minority stress oriented fears, such as ‘are you afraid of being outed by your partner’ or ‘what has your experience been like with law enforcement’ officials, yet current research demonstrates one form of psychological control and aggression in same-sex relationships surrounds being ‘outed’ and that there is greater reluctance to call the police during an incident of IPV due to law enforcement officials who have been shown greater rates of discrimination towards same-sex couples when answering a domestic violence call [25,27,33]. Likewise, a partner could threaten to disclose transgender status. In the United States, there is no federal law that prevents firing a lesbian or a trans person because she is a lesbian or trans person so this threat can wield a significant amount of control and is not assessed in any of the scales. Two scales address these issues better than the majority of existing measurement tools are the Conflict Tactics Scale (CTS) and the Lesbian Partner Abuse scale (LE-PAS-R). 

Among the available scales that measure IPV, two scales were selected to be reviewed more critically: CTS & LE-PAS-R. The CTS, short form (CTS2S) is a 20-item assessment tool shortened from the 80-item full CTS [34]. The goal of the CTS is to assess the tactics that are used by parents, children, couples etc. to control or abuse others. The CTS2S is a shortened version to assess couples and their tactics whether they are contributing to abuse or the victim of abuse. Examples of questions include, “I insulted or swore or shouted or yelled at my partner,” “I pushed, shoved, or slapped my partner,” “I punched or kicked or beat up my partner.” Respondents can answer: Once in the past year (1); twice in the past year (2); 3–5 times in the past year (3); 6–10 times in the past year (4); 11–20 times in the past year (5); more than 20 times in the past year (6); not in the past year, but it did happen before (7); and this never happened (8). The CTS2S scale is a relatively brief scale, the questions are gender neutral and relationship status also uses the neutral term “partner” but the questions and scoring are complicated and difficult to understand. In addition, there are no internal reliability scores since the instrument is not summed to create a total score [34].

The Lesbian Partner Abuse Revised Scale (LE-PAS-R) is a measurement tool specifically geared towards women in relationships [35]. The LE-PAS-R is a 25-item measurement tool with six factors: communication and social skills, substance abuse, intergenerational transmission of violence, fake illness, internalized homophobia, and status differentials. Example questions include: “my partner yells at me,” “drinks alcoholic beverages excessively,” “threatens to tell people, who do not know, that I am a lesbian,” and “is an angry drunk. Responses include never (1); very rarely (2); a little of the time (3); someone of the time (4); a good part of the time (5); and very frequently (6). The LE-PAS-R has strong reliability (r = 0.94) [35]. The scale may be useful for cisgender females in same-sex relationships but does not consider bisexual, pansexual or trans*women, which perpetuates continued invisibility in the research.

### 2.1. Conceptual Models for Sexual Minority Women

Unfortunately, little is known about the causality of IPV in SMW relationships. While there are conceptual models that address stage of change for survivors of IPV preparing to leave [36], this is a general model that includes all women, primarily those who are heterosexual but may include some participants who are in same-sex relationships. Currently, the only conceptual model of the relationship between general risk factors of IPV and partnered lesbian women has found that minority stress, anger and alcohol use have significant roles in perpetrating IPV and psychological aggression within lesbian relationships [13,37]. However, this does not take into consideration unpartnered lesbian women, women who identify as bisexual or pansexual, or trans*women. In fact, except the LE-PAS-R scale, there are no validated instrument measuring IPV in lesbian/queer identified relationships. [28,35] and a long and significant history of measurement issues in using standardized instruments when examining IPV and the LGBT community [38,39]. Current research, although increasing in frequency, relies on instruments missing the nuances of relationships between SMW [28,29].

### 2.2. Resilience Versus Posttraumatic Growth

There has been confusion in the literature about the difference between posttraumatic growth (PTG) and resilience [40,41]. Resilience is frequently identified as a personality trait where one resists the impact of the trauma; or conversely is seen as an adaptive response where someone rebounds to a pre-trauma level of functioning after they have ‘recovered’ from the trauma [42] whereas some theorists argue that posttraumatic growth (PTG) is a form of resilience, and some argue that PTG stands on its own, and needs to be a more important framework because it does not rely on individual personality or temperament characteristics [40,41,43,44]. Resilience is often measured as the absence of symptoms, whereas PTG is the presence of a positive outcome. Posttraumatic growth posits that someone does not return to their prior state of functioning because the process of surviving the trauma and the following period itself can be a transformative process and the person is different (and has experienced growth) because they have survived the trauma. In order to not place individual burden on marginalized populations, such as SMW, the authors argue that PTG should be the framework through which IPV is explored and consider PTG as a potential form of adjustment to traumatic events [40,45]. Minority stress theory suggests that holding significantly marginalized identities is in itself a stressor, and based on one’s experiences in the world, can be traumatic. When these identities are considered in relationship to IPV, what personal transformation may occur from surviving these traumas? Understanding what positive personal and worldview changes that can come with being a survivor may help empower sexual minority women and mitigate the impact of minority stress, which is important for behavioral health providers in terms of treatment planning and service provision. Also noteworthy is that IPV tends to occur over a long period of time, suggesting that it may be difficult to resolve the trauma due to its ongoing nature (Amanor-Boadu et al. [46,47,48]). In fact, Uloa et al. [49] state that this timeline keeps the trauma of IPV in its own particular category as compared to other traumas, such as a life-threatening illness, and thus needs to be examined through a different lens. The transformative aspect of PTG, which is not based on returning to a baseline, pre-trauma level of functioning may then be a more appropriate fit for analysis of IPV with SMW. These suggestions for research and implications for practice will be discussed in greater detail below.

## 3. Posttraumatic Growth

Posttraumatic growth was initially developed in the 1990s by Calhoun and Tedeschi, who defined PTG as the struggle with traumatic exposure, such as IPV, individuals may also experience tremendous personal growth in five particular domains: relating to others, personal strength, new possibilities, appreciation of life and spiritual change. This is not necessarily a universal experience for all survivors of trauma, but there can be this paradoxical effect that something initially experienced as negative can bring about positive psychological changes through a transformative process [45]. The framework of PTG was initially grounded in qualitative research and identified three domains. This was followed by the development of the quantitative measurement tool, the Posttraumatic Growth Inventory (PTGI). PTG does not deny that suffering occurs as part of experiencing a traumatic event and Calhoun and Tedeschi [50] are clear that the experience of PTG is not considered universal for all trauma survivors.

The PTGI [50] is a 21-item self-report inventory that measures an individual’s perception of positive change following a traumatic life experience. Items are rated on a scale from 0 (“I did not experience this change as a result of my crisis”) to 5 (“I experienced this change to a very great degree as a result of my crisis”). Responses to items are summed to produce a total PTG score which ranges from 0 to 126, with higher scores indicating higher posttraumatic growth. The PTGI indicates five domains of growth, or factors: (1) new possibilities (“I established a new path for my life”); (2) relating to others (“Knowing that I can count on people in times of trouble”); (3) personal strength (“Knowing I can handle difficulties”); (4) appreciation of life (“An appreciation for the value of my life”); and (5) spiritual change (“A better understanding of spiritual matters”). The overall PTGI has excellent internal consistency (a = 0.90) [51]. The five factors have acceptable to good internal consistency: new possibilities has 5 items (a = 0.84), relating to others has 7 items (a = 0.85), personal strength has four items (a = 0.72) appreciation of life has three items (a = 0.67) and spiritual change has two items (a = 0.85) [51].

### 3.1. Posttraumatic Growth and Intimate Partner Violence

While reviewing overall quantitative studies looking at outcomes of PTG and how it relates to IPV within heterosexual relationships, the limited results are variable. Valdez and Lilly [52] found in their study of PTG in female (heterosexual) survivors of IPV, and similar to earlier research conducted by Calhoun and Tedeschi [53], that 87% of their respondents reported PTG post-IPV related trauma found that women who experienced more abuse in their relationships experienced higher rates of posttraumatic growth. Doane [54] explored the role of timeline posttrauma and found that the greater the distance the participant had from the abusive relationship, the higher the scores on the PTGI, but this study looked at IPV survivors in comparison to violent crime survivors and did not focus on sexual minorities.

The use of PTG to more deeply understand the impact of IPV could also address another long-standing issue regarding research around the experience of IPV for all survivors, not just SMW. Extensive research on the effects of IPV inclusive of all women tend to fall into one of two categories. The first group consists of women who are concurrently experiencing, or have just recently experienced IPV during the data collection process and have been shown to have increased behavioral health symptoms including higher levels of anxiety, depression, substance use, and psychosocial stress [31,55] or longitudinal studies of the consequences of IPV on behavioral health for both the survivors of IPV themselves as well as the impact on children who experience IPV in the home [56,57,58]. Most research focuses on short-term impact or long-term effects of IPV, but there is a lack of clarity in current research delineating any differences between the intermittent experience or single-incident IPV versus longer-term IPV and further research on any possible distinction between the two is needed. The lens of PTG considers trauma as an experience whether it happens once or repetitively, and could provide a deeper understanding of which domains are the least, or conversely, most likely to manifest, particularly if there are differences between single/short-term IPV and longer term IPV. Not only do more inclusive and nuanced measurement tools need to be developed for SMW, but the lens through which survivors integrate their past traumatic experiences could benefit from the empowerment approach taken by PTG theory particularly for groups that experience multiple points of oppression and marginalization.

### 3.2. Posttraumatic Growth and Sexual Minority Women

Research focusing specifically on trauma and SMW has called for a deeper look into factors of resilience, especially the role of social support [59]. Models of resilience include conceptual frameworks such as stress-related growth (SRG) and posttraumatic growth (PTG). Bonet, Wells and Parsons [60] explored SRG as an aspect of the coming out process for lesbian and bisexual women, Putney, Leefmeaker and Herbert [61] have looked at aging lesbians, SRG and social support, while Golub, Walker et al. [62] explored SRG, social support and HIV risk for transgender women and Calabrese et al. [63] looked at SRG for SMW in terms of minority stress. Although stress-related growth touches on some of the same factors of personal improvement following stress, there are critical epistemological differences in that SRG encompasses developmental life stressors and everyday stressors, whereas PTG focuses on a traumatic event in which someone’s assumptive world is challenged and shattered [53,64]. 

For the purposes of this conceptual argument, intimate partner violence (IPV), single-episode, or ongoing violence, will be considered as a traumatic event. Trauma, as conceptualized by Calhoun and Tedeschi [50] is identified as a singular or repeated traumatic event, also known as a crisis or major stress, that is defined by very difficult circumstances that “significantly challenge or invalidate important components of the person’s assumptive world” and thus the posttraumatic framework should be explored when considering risk and protective factors in working with survivors of IPV. PTG has been identified as present with survivors of assault in particular areas of the five domains, including become a stronger person and identifying higher levels of empathy with others, yet there remains a gap in exploring PTG and IPV as a whole, particularly to seek understanding as to why growth may not be seen in some but not all five domains [52,65,66].

In terms of sexual minority communities, Calhoun and Tedeschi’s specific model of PTG has been explored with HIV-positive gay men [67,68] and with military veteran transgender elders and identity [69], yet significant gaps remain in terms of factors of PTG within SMW. Our study calls for deeper and more specific quantitative and qualitative research in exploring the relationship between IPV and the potential for PTG in sexual minority women.

## 4. Proposal/Implications

The authors propose using the PTG framework to analyze the phenomenon of how SMW may integrate the experience of IPV to move forward with their lives, particularly in order to develop more inclusive programs and services. The analysis of IPV through the lens of PTG would allow for the exploration of which of the five domains do SMW experience the most or, conversely, the least amount of PTG following the experience of surviving IPV. What role and what significance does minority stress play in understanding the experience of trauma and psychological growth when exploring risk factors and protective factors in same-sex relationships? One of the five domains of PTG is relation to/with others and as such, what role does social support exactly play in terms of relating to others when a sexual minority identity is factored in? How is this social support sought out and does it differ from those who hold dominant identities? Limited research demonstrates that SMW can have higher factors of resilience through social support within sexual minority communities [70,71], but resilience focuses more on aspects of individual temperament. How is social support sought out for women who have fled their residence to live in an emergency shelter, particularly if they have been ostracized by their family-of-origin for their sexual identity, or how might a trans*woman seek out social support in a shelter system designed around heterocentric norms? Another question for analysis would focus on the improved outcome of spirituality or spiritual activities for sexual minority women who demonstrate PTG after experiencing IPV, or is that a domain that is limited because sexual minorities often experience discrimination and/or exclusion in many worldwide religions?

For the domain of new possibilities, does sexual minority status and female-identified gender factor into how this domain may manifest as PTG? Or conversely, is the intersection of gender and sexual minority identity such an additional stressor with deep systemic roots so that new possibilities may be more limited for marginalized populations than for those with dominant identities. Minority stress may also impact the experience of the fourth domain: appreciation for life. Minority stress theory posits that a minority identity leads to increased stress, and has inherent risk factors, which may directly contribute to the experience of not appreciating one’s life due to holding a historically oppressed identity. And finally, does internalized heterosexism limit the belief in personal strength, the fifth factor, or does surviving multiple layers of oppression contribute to higher levels of improvement in this specific domain?

In addition to PTG as a lens through which to understand survivorship of SMW who experience IPV, the authors recommend the development of scales that speak to the nuanced experience of sexual minorities as a whole particularly in terms of language, as well as revisions to the Conflict Tactics Scale (CTS2S) and the Lesbian Partner Abuse Scale (LE-PAS-R) specifically for SMW. Both scales have strengths but need revision to be fully inclusive of SMW. The brief CTS2S scale has gender neutral questions but the complicated scoring makes it unlikely to be used by practitioners. The LE-PAS-R focuses on lesbian cisgender woman but does not consider bisexual, pansexual or trans*women. Revisions to these scales may be useful when examining the levels of IPV in SMW. Using both the PTGI and a measurement tool designed for IPV survivors would provide a deeper lens through which to understand the impact of IPV. The authors also call for a greater understanding of how multiple systemic factors such as race, geographic locale, socioeconomic status and social support may influence the presence of posttraumatic growth following the experience of IPV. This could include how these factors may facilitate or hinder the growth of PTG and have practice as well as program development implications for emergency shelters, law enforcement officials, medical practitioners, and behavioral health care providers.

## 5. Conclusions

By understanding the intersection of minority stress, intimate partner violence and the possibility of posttraumatic growth, stronger programs in emergency shelters and in inpatient and outpatient counseling programs could be developed and implemented that are tailored to the nuanced needs of SMW. Deeper understanding of how individuals and marginalized groups can adapt positively from experienced trauma answers Valdez & Lilly’s recent [52] call for a more comprehensive framework for working with trauma survivors. Through a clearer understanding of this posttrauma adaptation, more nuanced programming that fosters these domains may prevent future behavioral health risk factors for survivors and increase the ability to not only survive, but thrive. Knowledge as to how PTG can be facilitated for all survivors of IPV would make a significant contribution to better emotional and behavioral health outcomes and has the ability to strengthen the curriculum of both policy-based and clinical social work programs. The intersection of these variables viewed through a lens that specifically takes into consideration the unique stressors faced by sexual minority women would allow for more reliable data to be gathered so that more sustainable and tailored programs and interventions could be developed so that more positive outcomes may be perpetuated.

## References

[1] Barrett B.J., St. Pierre M. (2013). Intimate partner violence reported by lesbian-, gay-, and bisexual-identified individuals living in Canada: An exploration of within-group variations. J. Gay Lesbian Soc. Serv..

[2] Department of Reproductive Health and Research, World Health Organization (2013). Global and Regional Estimates of Violence against Women: Prevalence and Health Effects of Intimate Partner Violence and Non-Partner Sexual Assault. www.who.int/reproductivehealth.

[3] Tjaden P., Thoennes N. (2000). Extent, Nature, and Consequences of Intimate Partner Violence: Findings from the National Violence against Women Survey (Report No. NCJ 181867).

[4] Smith S.G., Chen J., Basile K.C., Gilbert L.K., Merrick M.T., Patel N., Walling M., Jain A. (2017). The National Intimate Partner and Sexual Violence Survey (NISVS): 2010–2012 State Report.

[5] Nathanson A.M., Shorey R.C., Tirone V., Rhatigan D.L. (2012). The prevalence of mental health disorders in a community sample of female victims of intimate partner violence. Partn. Abus..

[6] Shorey R., Tirone V., Nathanson A., Handsel V., Rhatigan D. (2012). A preliminary investigation of the influence of subjective norms and relationship commitment on stages of change in female intimate partner violence victims. J. Interpers. Violence.

[7] Frankland A., Brown Z. (2014). Coercive control in same-sex intimate partner violence. J. Fam. Violence.

[8] Gehring K., Vaske J. (2017). Out in the open: The consequences of intimate partner violence for victims in same-sex and opposite sex relationships. J. Interpers. Violence.

[9] Sorenson S., Thomas K. (2009). Views of intimate partner violence in same- and opposite sex relationships. J. Marriage Fam..

[10] Baker N., Buick J., Kim S., Moniz S., Nava K. (2013). Lessons from examining same-sex intimate partner violence. Sex Roles.

[11] Meyer I.H. (2003). Prejudice, social stress, and mental health in lesbian, gay, and bisexual populations: Conceptual issues and research evidence. Psychol. Bull..

[12] Lewis R., Kholodkov T., Derlega V. (2012). Still stressful after all these years: A review of lesbians’ and bisexual women’s minority stress. J. Lesbian Stud..

[13] Lewis R., Mason T., Winstead B., Kelley M. (2017). Empirical investigation of a model of sexual minority specific and general risk factors for intimate partner violence among lesbian women. Psychol. Violence.

[14] DiPlacido J., Herek G.M. (1998). Minority Stress among Lesbians, Gay Men, and Bisexuals: A Consequence of Heterosexism, Homophobia, and Stigmatization. Stigma and Sexual Orientation.

[15] Williamson I.R. (2000). Internalized homophobia and health issues affecting lesbians and gay men. Health Educ. Res..

[16] Everett B. (2015). Sexual orientation identity change and depressive symptoms. J. Health Soc. Behav..

[17] Hatzenbuehler M.L., Hilt L.M., Nolen-Hoeksema S., Chrisler J., McCreary D. (2010). Gender, sexual orientation, and vulnerability to depression. Handbook of Gender Research in Psychology.

[18] Lehavot K., Simoni J.M. (2011). The impact of minority stress on mental health and substance use among sexual minority women. J. Consult. Clin. Psychol..

[19] Miller B., Irvin J. (2017). Invisible scars: Comparing the mental health of LGB and heterosexual intimate partner violence survivors. J. Homosex..

[20] Pyra M., Weber K.M., Wilson T.E., Cohen J., Murchison L., Goparaju L., Cohen M.H. (2014). Sexual minority women and depressive symptoms throughout adulthood. Am. J. Public Health.

[21] Farmer G., Blosnich J., Jabson J., Matthews D. (2015). Gay Acres: Sexual Orientation Differences in Health Indicators among Rural and Non-rural Individuals. J. Rural Health.

[22] Whitehead J., Shaver J., Stephenson R. (2016). Outtness, stigma, and primary health care utilization among rural LGBT populations. PLoS ONE.

[23] Murray C., Mobley K. (2009). Empirical Research about Same-Sex Intimate Partner Violence: A Methodological Review. J. Homosex..

[24] (2006). National Coalition of Anti-Violence Lesbian, Gay, Bisexual and Transgender Domestic Violence in the United States in 2006. http://www.ncdsv.org/images/NCAVP_LBGTDVin2006.pdf.

[25] Carvalho A., Lewis R., Derlega V., Winstead B., Viggiano C. (2011). Internalized sexual minority stressors and same-sex intimate partner violence. J. Fam. Violence.

[26] West C. (2012). Partner abuse in ethnic minority and gay, lesbian, bisexual and transgender populations. Partn. Abus..

[27] Murray C., Mobley A., Buford A., Seaman-DeJohn M. (2006). Same-sex intimate partner violence: Dynamics, social context, and counseling implications. J. LGBT Issues Couns..

[28] Mason T., Lewis R., Milletich R., Kelley M., Minifie J., Derlega V. (2014). Psychological aggression in lesbian, gay, and bisexual individuals’ intimate relationships: A review of prevalence, correlates, and measurement issues. Aggress. Violent Behav..

[29] Rausch M. (2016). Adverse childhood experiences and intimate partner violence in lesbian and queer relationships. J. LGBT Issues Couns..

[30] Dutton M.A., Holtzworth-Munroe A., Jouriles E., McDonarld R., Krishnan S., McFarlane J. (2003). Recruitment and Retention in Intimate Partner Violence Research (NCJ 201943).

[31] Golding J.M. (1999). Intimate partner violence as a risk factor for mental disorders: A meta-analysis. J. Fam. Violence.

[32] Lindhorst T., Beadnell B. (2011). The long arc of recovery: Characterizing intimate partner violence and its’ psychosocial effects across 17 years. Violence Women.

[33] Ristock J. (2005). Relationship Violence in Lesbian/Gay/Bisexual/Transgender/Queer [LGBTQ] Communities: Moving beyond a Gender-Based Framework. Violence Women Online Resources. http://www.incava.umn.edu/documents/lgbtqviolence/lgbtqviolence.pdf.

[34] Straus M., Douglas E. (2004). A short form of the revised conflict tactics scales and typologies for severity and mutuality. Violence Vict..

[35] McClennen J., Summers A., Daley J. (2002). The Lesbian Partner Abuse Scale. Res. Soc. Work Pract..

[36] Bouhnik D. (2007). A model design proposal of a supportive website for women experiencing IPV. J. Inf. Commun. Ethics Soc..

[37] Calton J., Catteneo L., Gebhard K. (2015). Barriers to help seeking for lesbian, gay, bisexual, transgender, and queer survivors of intimate partner violence. Trauma Violence Abus..

[38] Follingstad D.R. (2007). Rethinking current approaches to psychological abuse: Conceptual and methodological issues. Aggress. Violent Behav..

[39] Jordan C.E., Campbell R., Follingstad D. (2010). Violence and women’s mental health: The impact of physical, sexual, and psychological aggression. Annu. Rev. Clin. Psychol..

[40] Bensimon M. (2012). Elaboration on the association between trauma, PTSD and post-traumatic growth: The role of trait resilience. Pers. Indiv. Differ..

[41] Tedeschi R.G., Calhoun L.G., Cann A. (2007). Evaluating resource gain: Understanding and Misunderstanding posttraumatic growth. Appl. Psychol..

[42] Lazarus R.S. (1993). From psychological stress to emotions—A history of changing outlooks. Annu. Rev. Psychol..

[43] Agaibi C.E., Wilson J.P. (2005). Trauma, PTSD, and resilience. A review of the literature. Trauma Violence Abus..

[44] Lepore S.J., Revenson T.A., Calhoun L.G., Tedeschi R.G. (2006). Resilience and posttraumatic growth: Recovery, resistance, and reconfiguration. Handbook of Posttraumatic Growth: Research and Practice.

[45] Tedeschi R.G., Calhoun L.G. (2004). Posttraumatic growth: Conceptual foundations and empirical evidence. Psychol. Inq..

[46] Amanor-Boadu Y., Messing J., Stith S.M., Anderson J.R., O’Sullivan C.S., Campbell J.C. (2012). Immigrant and nonimmigrant women: Factors that predict leaving an abusive relationship. Violence Women.

[47] Choice P., Lamke L.K. (1999). Stay/leave decision-making processes in abusive dating relationships. Pers. Relatsh..

[48] Hamby S.L., Gray-Little B., Kendall-Tackett K., Giacomoni S. (2007). Can battered women cope? A critical analysis of research of women’s responses to violence. Intimate Partner Violence.

[49] Uloa E., Hammett J., Guzman M., Hokoda A. (2015). Psychological growth in relation to intimate partner violence: A review. Aggress. Violent Behav..

[50] Tedeschi R.G., Calhoun L.G. (1996). The posttraumatic growth inventory: Measuring the positive legacy of trauma. J. Trauma. Stress.

[51] Taku K., Cann A., Calhoun L., Tedeschi R. (2008). The factor structure of the posttraumatic growth inventory: A comparison of five models using confirmatory factor analysis. J. Trauma. Stress.

[52] Valdez C., Lilly M. (2015). Post-traumatic growth in survivors of intimate partner violence: An assumptive world process. J. Interpers. Violence.

[53] Calhoun L.G., Tedeschi R.G. (2006). The Handbook of Posttraumatic Growth: Research and Practice.

[54] Doane N.K. (2011). Predictors of Post-Traumatic Growth, Shame, and Post-Traumatic Stress Symptoms in Survivors of Intimate Partner Violence: The Roles of Social Support and Coping. Ph.D. Thesis.

[55] Roberts G.L., Williams G.M., Lawrence J.M., Raphael B. (1998). How does domestic violence affect women’s mental health?. Women Health.

[56] Anderson D.K., Saunders D.G., Yoshihama M., Bybee D.I., Sullivan C.M. (2003). Long-term trends in depression among women separated from abusive partners. Violence Women.

[57] Holmes M. (2013). The sleeper effect of intimate partner violence exposure: Long-term consequences on young children’s aggressive behavior. J. Child Psychol. Psychiatry.

[58] Narayan A., Labella M., Englund M., Carlson E., Egeland B. (2017). The Legacy of Early Childhood Violence Exposure to Adulthood Intimate Partner Violence: Variable- and Person-Oriented Evidence. J. Fam. Psychol..

[59] Balsam K.F., Molina Y., Blayney J.A., Dillworth T., Zimmerman L., Kaysen D. (2015). Racial/ethnic differences in identity and mental health outcomes among young sexual minority women. Cult. Divers. Ethn. Minor. Psychol..

[60] Bonet L., Brooke E., Wells M., Parsons J. (2007). A positive look at a difficult time: A strengths-based examination of coming out for lesbian and bisexual women. J. LGBT Health Res..

[61] Putney J., Leafmeeker R., Herbert N. (2016). “The wisdom of age”: Perspectives on aging and growth among Lesbian Older Adults. J. Gerontol. Soc. Work.

[62] Golub S., Walker J., Longmire-Avital B., Bimbi D., Parsons J. (2010). The role of religiosity, social support, and stress-related growth in protecting against HIV risk among transgender women. J. Health Psychol..

[63] Calabrese S., Meyer I., Overstreet N., Haile R., Hansen N. (2014). Exploring discrimination and mental health disparities faced by black sexual minority women using a minority stress framework. Psychol. Women Q..

[64] Joseph S., Butler L.D. (2010). Positive changes following adversity. Posttraum. Stress Disord. Res. Q..

[65] Frazier P., Conlon A., Glaser T. (2001). Positive and negative life changes following sexual assault. J. Consult. Clin. Psychol..

[66] Shakespeare-Finch J., De Dassel T. (2009). Exploring posttraumatic outcomes as a function of childhood sexual abuse. J. Child Sex. Abus..

[67] Eggleston J.J. (2015). Posttraumatic Growth in People Living with HIV/AIDS: Psychological, Spiritual and Physical Health-Related Outcomes. ETD Collection for Fordham University. AAI3719352. http://fordham.bepress.com/dissertations/AAI3719352.

[68] Yu M., Chen L., Ye Z., Li X., Lin D. (2017). Impacts of making sense of adversity on depression, posttraumatic stress disorder, and posttraumatic growth among a sample of mainly newly diagnosed HIV-positive Chinese young homosexual men: The mediating role of resilience. AIDS Care.

[69] Hoy-Ellis C., Shiu C., Sullivan K., Kim H., Sturges A., Fredriksen-Goldsen K. (2017). Prior military service, identity stigma, and mental health among transgender older adults. Gerontologist.

[70] Kwon P. (2013). Resilience in lesbian, gay and bisexual individuals. Pers. Soc. Psychol. Rev..

[71] Zimmerman L., Darnell D.A., Rhew I.C., Lee C.M., Kaysen D. (2015). Resilience in community: A social ecological development model for young adult sexual minority women. Am. J. Community Psychol..

